# Canonical and Divergent N-Terminal HBx Isoform Proteins Unveiled: Characteristics and Roles during HBV Replication

**DOI:** 10.3390/biomedicines9111701

**Published:** 2021-11-16

**Authors:** Sergio Hernández, Francisca Álvarez-Astudillo, Daniel Garrido, Cristian Prieto, Alejandra Loyola, Rodrigo A. Villanueva

**Affiliations:** 1 Centro Ciencia & Vida, Fundación Ciencia & Vida, Avda. Zanartu 1482, Nunoa, Santiago 7780272, Chile; zenden21@gmail.com (S.H.); fca.alvarez@gmail.com (F.Á.-A.); daniel.garrido.n@gmail.com (D.G.); cr.prietop@gmail.com (C.P.); 2 Facultad de Medicina y Ciencia, Universidad San Sebastian, Santiago 7510157, Chile

**Keywords:** hepatitis B virus, HBV, hepatitis B virus X protein, HBx, subcellular localization, localization regulation, genome replication, divergent N-terminal isoform

## Abstract

Hepatitis B virus (HBV) X protein (HBx) is a viral regulatory and multifunctional protein. It is well-known that the canonical HBx reading frame bears two phylogenetically conserved internal in-frame translational initiation codons at Met2 and Met3, thus possibly generating divergent N-terminal smaller isoforms during translation. Here, we demonstrate that the three distinct HBx isoforms are generated from the ectopically expressed HBV HBx gene, named XF (full-length), XM (medium-length), and XS (short-length); they display different subcellular localizations when expressed individually in cultured hepatoma cells. Particularly, the smallest HBx isoform, XS, displayed a predominantly cytoplasmic localization. To study HBx proteins during viral replication, we performed site-directed mutagenesis to target the individual or combinatorial expression of the HBx isoforms within the HBV viral backbone (full viral genome). Our results indicate that of all HBx isoforms, only the smallest HBx isoform, XS, can restore WT levels of HBV replication, and bind to the viral mini chromosome, thereby establishing an active chromatin state, highlighting its crucial activities during HBV replication. Intriguingly, we found that sequences of HBV HBx genotype H are devoid of the conserved Met3 position, and therefore HBV genotype H infection is naturally silent for the expression of the HBx XS isoform. Finally, we found that the HBx XM (medium-length) isoform shares significant sequence similarity with the N-terminus domain of the COMMD8 protein, a member of the copper metabolism MURR1 domain-containing (COMMD) protein family. This novel finding might facilitate studies on the phylogenetic origin of the HBV X protein. The identification and functional characterization of its isoforms will shift the paradigm by changing the concept of HBx from being a unique, canonical, and multifunctional protein toward the occurrence of different HBx isoforms, carrying out different overlapping functions at different subcellular localizations during HBV genome replication. Significantly, our current work unveils new crucial HBV targets to study for potential antiviral research, and human virus pathogenesis.

## 1. Introduction

Hepatitis B virus (HBV) infection is a major health problem affecting millions of people globally. Even though the administration of an effective prophylactic vaccine has significantly reduced new cases since the 1980s, a therapeutic cure for chronic hepatitis B is still unavailable. HBV acute infection can progress to chronic disease and liver damage such as cirrhosis, liver failure, and hepatocellular carcinoma (HCC). In 2015, the WHO estimated that 257 million individuals are chronically infected with HBV, corresponding to 3.5% of the global population [1]. Thus, chronic infection by HBV is considered a global public health catastrophe with millions of people affected [2].

HBV is a small, enveloped DNA virus and a member of the *Hepadnaviridae* family. The HBV genome is a partially double-stranded, circular DNA molecule of 3.2 kb. Upon infection, the viral genome is repaired, forming the viral intermediate covalently closed circular DNA, cccDNA, which is kept in the nucleus of the infected hepatocyte and is responsible for persistent infection [3]. Ten genotypes have been identified for HBV: genotypes A to J [4]. The HBV genotypes show different geographical distributions. Genotypes F and H are autochthonous and prevalent in Latin America [5]. Genotype F also circulates in Alaska where HBV subgenotype F1b is prevalent. The genetic variability of the different HBV isolates is related to disease outcomes [6]. This is particularly so for HBV genotype F, which is associated with early, frequent, and rapid progression to HCC [7,8,9,10,11]. In contrast, infection with HBV genotype H, circulating in Mexico, is characterized by low endemicity, low viral load, few cases of acute and chronic liver diseases, and a low prevalence of HCC [12,13]. Patients infected with the HBV genotype H are usually asymptomatic without clinical manifestations of liver disease [14,15]. In addition, HBV genotype H infection has a high prevalence of occult hepatitis B infection (OBI), which is defined by the low presence of circulating HBV DNA in either serum or liver tissue in patients who were diagnosed negative for HBsAg [14]. The molecular determinants that differentiate the HBV infection outcome of these two genotypes are unknown.

**Figure 1:**
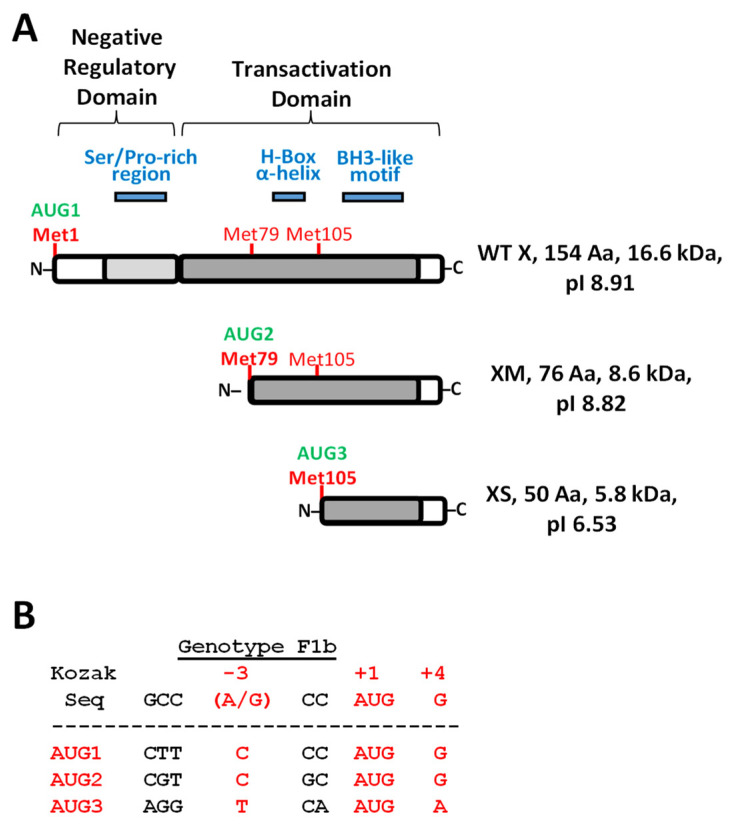

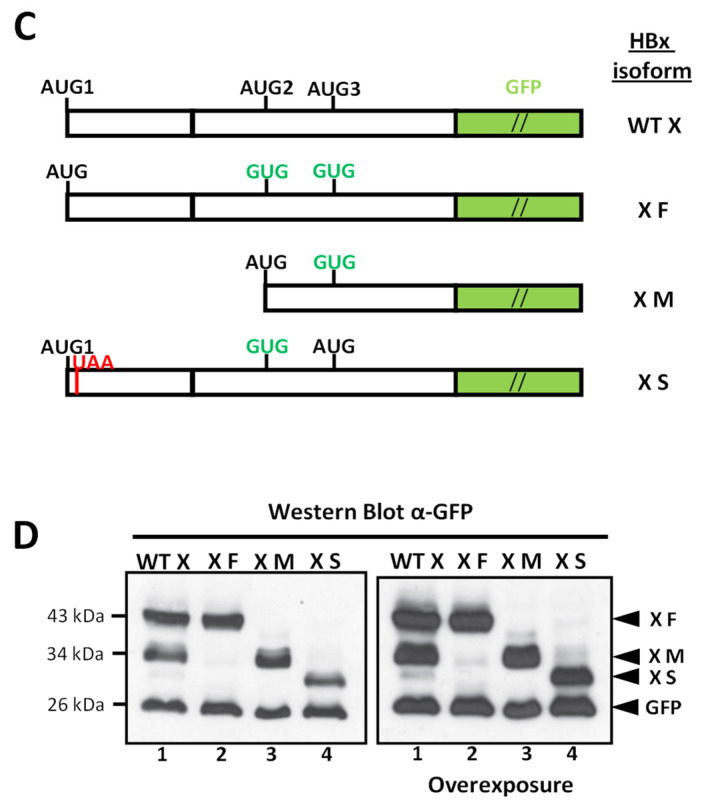
HBx domains organization, smaller isoforms, and their constructs for expression in Huh-7 hepatocarcinoma cells. (**A**) HBx functional domains organization [22,23,24,25,26]. N-terminal negative regulatory domain includes the AUG1 (green), Met1 (bold red), and Ser/Pro-rich region segment (blue). HBx C-terminal transactivation domain with AUG2 (green), Met79 for HBx Medium smaller isoform (XM), including H-box α-helix (blue). AUG3 (green), Met105 for HBx smaller isoform (XS), including the BH3-like motif (blue) are shown. Calculated molecular weight and basal isoelectric point (pI) for each HBx-derived isoform polypeptides are indicated to the right. (**B**) HBx in-frame AUG1, AUG2, and AUG3 initiation codons of HBV genotype F1b (Genebank KM233681.1) [27,28] compared with Kozak consensus sequences. Critical positions (−3, and +4) for optimal translation with respect to Kozak consensus are shown in red. (**C**) Scheme of HBx constructs for either canonical or individual HBx isoforms expression fused to the GFP marker. HBx constructs were made with the indicated regions of the HBx reading frame (white blocks), and with the substituted point changes (AUG -> GUG). For HBx XS expression (“mini-HBx”), the complete HBx reading frame was necessary for expression, introducing an in-frame early stop codon to prevent full HBx expression (UAA and red line) and a GUG change in the AUG2 to prevent the expression of XM. (**D**) Expression of the HBx isoforms upon transfection in Huh-7 cells, and Western blot to detect GFP expression. DNA constructs were transfected in Huh-7 cells, cell extracts were resolved by SDS-PAGE, and HBx isoform proteins were detected by Western blot against GFP protein marker. WT HBx (WT X, lane 1), HBx full-length (XF, lane 2), HBx medium-length (XM, lane 3), and HBx small-length (XS, lane 4) are indicated. Right panel is an overexposure of the signal showed to the left.

The HBx protein is encoded by the smallest HBV gene and is the only known viral regulatory protein. The canonical HBx protein is essential for viral replication and HBV infection [16,17,18,19,20,21]. The full-length canonical HBx protein is composed of 154 residues that are organized into two distinct functional domains (Figure 1A) [22,23,24,25,26].

The N-terminal domain (residues 1 to 50) is composed of a highly conserved region displaying transrepressor activity (residues 21–50) [29] as well as a Ser/Pro-rich region [23]. Importantly, the HBx N-terminal residues 1 to 42 are dispensable for replication [30]. On the other hand, the C-terminal region (residues 52 to 142) contains a transactivation domain, which is required for transcriptional transactivation and enhancement of HBV genome replication [31,32]. Further mapping analyses of the C-terminal domain showed that the region comprising residues 58 to 119 is involved in signal transduction activities [33], region residues 120–140 in nuclear transactivation mechanisms [34,35], and the last 20 residues in HBx stability [35]. The negative regulatory domain represses the transactivation activity of the HBx C-terminal domain, and thus represses HBx transactivation (Figure 1A) [22]. Furthermore, the C-terminal domain of HBx contains sequences important for mitochondrial localization [36,37]. The 3D structure of viral HBx is unknown. The N-terminus bears a highly conserved but unstructured and disordered region [38]. On the other hand, the HBx C-terminal is more structured [39] with a proposed zinc finger motif [40], a conserved alpha-helix (residues 88 to 100), the H-box [41], and a BH3-like motif (residues 113–135) [42,43] (Figure 1A). Thus, for canonical HBx, most of the multifunctional activities reside within its C-terminal domain whereas the N-terminal region is apparently not essential for viral protein function.

There is no consensus on the HBx subcellular localization as some in vitro and liver biopsy analyses have reported that it is preferentially cytoplasmic [44,45,46,47]; others have shown it to be a nuclear protein [48,49]. However, this discrepancy might be because HBx subcellular localization is influenced by its relative abundance. Thus, when HBx is expressed at low levels, it is predominantly nuclear, but it accumulates in the cytoplasm when expressed at higher levels [50,51]. This differential nuclear/cytoplasmic localization might explain the numerous functions of the HBx protein [52,53]. In the cytoplasm, HBx can alter mitochondria metabolism, apoptosis, and a variety of signal transduction cascades [17,54,55,56,57,58]. In the nucleus, HBx transactivates a diverse array of viral and cellular promoters [59]. Nuclear HBx is required due to its functional role in viral replication [60]. Given that HBx does not directly bind to dsDNA [61], its ability to activate the transcription of host genes is thought to proceed via protein–protein interactions with nuclear proteins such as transcription factors [23,62,63,64,65,66]. Multiple DNA repair pathways are affected by the expression of HBx [67]. Together, these findings suggest that HBx expression may play a role in cell pathogenesis and development of HCC, by targeting different cellular pathways at different subcellular locations.

Alternative translation initiation (ATI) is a mechanism by which a single mRNA results in the translation of protein isoforms with different N-termini due to more than one initiation codon in the same reading frame [68,69,70]. The ATI mechanism diversifies the proteome, altering function and/or subcellular localization of proteins [70]. HBV gene expression is regulated by four promoters and two enhancers, leading to the production of six distinct mRNAs, including the 0.7-kb HBx mRNA [26]. Transcription of the canonical HBx is regulated by the X promoter, located upstream of the transcription initiation site. The minimal promoter sequence overlaps the 3′ end of the enhancer I [71,72,73]. The viral HBx mRNA bears two internal in-frame translation initiation codons at the positions of AUG2 and AUG3 as detailed in Figure 1B. These positions are highly conserved across HBV genotypes. Alternative translation initiation from these two internal AUG codons would produce two additional divergent N-terminal smaller HBx isoforms (Figure 1A,B). Mutational analyses of the in-frame internal AUG codons have provided some evidence that divergent N-terminus HBx isoforms are produced by ATI [74]. Moreover, the HBx protein isoforms were found to be functionally different [75,76]. However, these analyses were performed in heterologous systems, using ectopic expression of the HBx ORF in cultured cells. Recently, a smaller HBx isoform protein was detected by ribosomal profiling of HBV-replicating cells corresponding to the HBx isoform initiated at AUG2 (Figure 1A,B). This isoform displayed a different function than that of the canonical HBx [77].

After infection, the HBV virion DNA is transformed to cccDNA, which serves as the template for all viral transcription [78]. In the nucleus, HBV cccDNA is organized as a mini chromosome forming the typical “beads-on-a-string” structure of cellular chromatin, with regularly spaced nucleosomes containing histone and non-histone proteins [79,80]. HBcAg and HBx viral proteins bind to the cccDNA mini chromosome, altering its structure [81]. Many groups, including our group, have characterized how post-translational modifications on the histones associated with cccDNA regulate viral transcription [82,83,84,85,86,87,88,89]. Histone methylation of arginine 3 on histone H4, methylation of lysines 9 and 27 on histone H3, and hypoacetylation of histones are all modifications that correlate with repressed transcription, while histone methylation of lysine 4 on histone H3 and hyperacetylation of histones H3 and H4 correlate with active transcription [89]. Consistently, chromatin-modifying enzymes that establish modifications on histone proteins are recruited to the cccDNA [82,84,85,86,89]. Histone variants also regulate HBV viral transcription, as illustrated by H3.3, which is assembled into the cccDNA and activates transcription [90]. Importantly, the HBx protein modulates the cccDNA chromatin landscape by regulating the recruitment of chromatin-modifying enzymes [82,85,86,87,91,92,93,94]. Indeed, when HBx protein is present, cccDNA is in an active chromatin state that promotes HBV transcription, genome replication, and production of viral progeny [82,84,86]. In contrast, in the absence of HBx, the cccDNA is in an inactive chromatin state [21,82,84,87]. Finally, it has been proposed that HBV HBx-deficient infection resembles the occult HBV infection observed in clinical cases: low transcriptional activity and persistence of the viral DNA [95], suggesting that the cccDNA is in an inactive chromatin state, as indicated. It is worth noting that infection by HBV genotype H has a high prevalence of occult HBV infection. Thus, it would be interesting to investigate the chromatin landscape of genotype H. All experiments so far have studied the canonical HBx protein; the role of smaller HBx isoforms on the regulation of cccDNA has not yet been investigated.

Since HBx protein is the only nonstructural and regulatory protein of HBV, the protein has been intensively investigated, utilizing HBV X-deficient constructs in several cellular and animal model systems, using either viral DNA transfection or HBV virus infection [16,17,18,19,20,21,25,44,60,82,96,97,98]. However, the requirement for HBx during HBV replication strongly depends on the assay and model system utilized, and thus the results have been variable. Commonly, to abolish the expression of HBx protein within the HBV backbone, a premature termination codon is engineered in the eighth codon of the HBx reading frame [96]. Based on the information presented above, this site-directed mutagenesis only abolishes the expression of the full-length canonical HBx protein (from AUG1), whereas both in-frame positions, AUG2 and AUG3, remain intact, and can direct for the synthesis of two divergent N-terminal smaller HBx isoforms from the same reading frame (Figure 1A,B). Herein, we have performed systematic site-directed mutagenesis to target the individual expression of the different divergent N-terminal HBx isoforms within the HBV viral backbone and assayed them during HBV replication. Our results unveil the different roles of HBx isoforms both in the presence or absence of the full-length HBx protein. We anticipate that our results will change the paradigm of HBx from being a unique canonical protein to becoming multiple N-terminal divergent HBx protein isoforms with overlapping roles during HBV replication. Notably, our current data unveil new crucial HBV targets to study for potential antiviral research, and human virus pathogenesis.

## 2. Materials and Methods

### 2.1. Antibodies

GFP (Abcam (#ab290) Waltham, MA, USA), histone H3 (Abcam (#ab1791) Waltham, MA, USA), H3K4me3 (Abcam (#ab8580) Waltham, MA, USA), H3K9me2 (Abcam (#ab1220) Waltham, MA, USA), anti-FLAG beads (Sigma Aldrich (#A2220), St. Louis, MO, USA).

### 2.2. Site-Directed Mutagenesis

The HBx reading frame (into AcGFP vector, Clontech, Takara Bio Inc., Mountain View, CA, USA) or the full-length HBV genome (into the TOPO XL PCR vector, LifeTechnologies Corp., Carlsbad, CA, USA) were used to generate the mutants. Corresponding plasmids were incubated with a pair of oligonucleotides (Supplementary Materials, Tables S1 and S2), and site-directed mutagenesis was performed with the QuikChange II kit (Agilent Technologies Inc., Santa Clara, CA, USA). DNA sequences of the selected clones were confirmed from the two strands of the plasmid DNAs.

### 2.3. Cell Lines, Cell Culture, and Transient Transfections

Huh-7 and HepG2 human hepatocarcinoma cell lines were grown and maintained in Dulbecco’s modified Eagle’s medium (DMEM), supplemented with 10% fetal bovine serum (FBS), 100 U/mL penicillin (Hyclone), 100 ug/mL Streptomycin, and 2 mM glutamine. The cells were incubated at 37 °C and 5% CO_2_, harvested by trypsinization and collected in complete DMEM before being seeded for experiments [27,28,86,99]. For expression of HBx isoforms fused to GFP, Huh-7 cells at 70% confluency were transfected with pAcGFP plasmids containing HBx either wild type, XF, XM, or XS isoforms with Lipofectamine 2000 (Life Technologies). Twenty-four hours post-transfection, cells were lysed with RIPA buffer (150 mM NaCl, 1 % NP-40, 0.1 % SDS, 50 mM Tris pH 8.0, protease inhibitor cocktail (Roche)), and analyzed by Western blot [53]. For fluorescence imaging of cells, all transient transfections to express HBx isoform proteins fused to GFP were carried out in Huh-7 cells using Lipofectamine 2000 (Life Technologies Corp., Carlsbad, CA, USA) with a ratio of DNA (µg): Lipofectamine (µL) = 1: 2.5 as previously reported [52]. In brief, for expression, three different amounts of plasmid DNA were utilized, corresponding to low (0.64 µg), medium (1.6 µg), or high (4 µg) for a standard 100 mm tissue culture dish, and cells at 70% confluency at the transfection [53]. All transient transfection reactions were carried out with Opti-MEM (Gibco, Life Technologies Corp. Carlsbad, CA, USA) media. At 6-h post-transfection, the media was changed and replaced by complete media until cells were processed. On the other hand, full length HBV genotype F genome (KM233681.1) [28] was released by SapI (Fermentas, Thermo Fisher Scientific, Waltham, MA, USA) digestion from the plasmid vector. After digestion, the 3.2 kb DNA was purified from agarose with Wizard SV Gel and PCR Clean-Up System (Promega Corp., Madison, WI, USA). HepG2 human hepatocarcinoma cells were seeded at a density of 1.5 × 10^6^ cells in 100 mm plates and transfected with 4 µg of HBV DNAs using X-tremeGENE™ HP DNA transfection reagent (Roche, Basel, Switzerland). Cells were harvested and analyzed after transfection as indicated for each experiment. Post-transfection supernatants were taken 72 h after transfection with either wild type or mutant HBV genomes. Samples were analyzed by ARCHITECT i1000 (Abbott Laboratories, Chicago IL, USA) to detect either HBsAg or HBeAg, obtaining a signal-to-cutoff ratio as reported [27,86].

### 2.4. Purification and Analysis of HBV Cytoplasmic Intermediates and cccDNA

HBV intermediates were purified as previously reported [27,86,90]. In brief, transfected cells were lysed with lysis buffer (10 mM Tris-Cl, pH 7.5; 50 mM NaCl; 1 mM EDTA; 0.5% Nonidet P40) in the presence of proteinase inhibitors for 4 min on ice. Nuclei were sedimented by centrifugation at 2400× *g* for 10 min. The supernatant was treated with 300 U DNase I for 1 h at 37 °C. Proteins were digested with 0.4 U of proteinase K (New England Biolabs, Ipswich, MA, USA) at 37 °C overnight and nucleic acids were purified by phenol-chloroform (1:1) extraction and ethanol precipitation. The nuclei were disintegrated by vortexing for 30 sec in nuclei lysis buffer (100 mM NaOH; 6% SDS), followed by incubation for 30 min at 37 °C. Sodium acetate was added to a final concentration of 600 mM and the pellet was discarded after centrifugation at 9600× *g* for 20 min. The cccDNA was purified twice by phenol-chloroform (1:1) extraction and then precipitated with ethanol. HBV intermediates were analyzed by real-time PCR (KAPA SYBR FAST, Universal qPCR kit) (Supplementary Materials, Table S3).

### 2.5. cccDNA Chromatin Immunoprecipitation Assays (ChIP)

Chromatin immunoprecipitation was performed as previously reported, with modifications [27,86,90]. In brief, transfected cells were crosslinked for 10 min with 1% formaldehyde at room temperature. The reaction was quenched with glycine at a final concentration of 125 mM. Then, the cells were washed twice with 1× PBS, resuspended in lysis buffer (5 mM Hepes, pH 8.0; 85 mM KCl; 1% Triton X-100 and proteinase inhibitors) and homogenized with a Dounce homogenizer 10 times using a loose pestle. The cell extract was collected by centrifugation at 5400× *g* for 1 min at 4 °C, resuspended in nuclei buffer (50 mM Tris-HCl, pH 8.0; 10 mM EDTA; 1% SDS and protease inhibitors) and incubated for 10 min on ice. IP dilution buffer (20 mM Tris-HCl, pH 8.0; 2 mM EDTA; 50 mM NaCl; 1% Triton X-100; 0.1% SDS and protease inhibitors) was added and chromatin was sheared at high power for 4 pulses of 5 min in a Bioruptor water bath sonicator (Diagenode Inc., Denville, NJ, USA) to obtain fragments of 400 bp or smaller, and centrifuged twice at 16,000× *g* for 10 min at 4 °C. Supernatant was collected and pre-cleared by incubating with 2 μg of IgG and 20 µL of protein A (Merck Millipore, Burlington, MA, USA) for 2 h at 4 °C with rotation. The supernatant was immunoprecipitated with specific antibodies for 12–16 h at 4 °C. Immunocomplexes were recovered with the addition of 20 μL of protein A or G (for rabbit or mouse IgG, respectively) agarose beads and 1 h incubation with rotation at 4 °C. Immunoprecipitated complexes were washed once with sonication buffer (50 mM Hepes, pH 7.9; 140 mM NaCl; 1 mM EDTA, pH 8.0; 1% Triton X-100; 0.1% sodium deoxycholate; 1% SDS), twice with LiCl buffer (100 mM Tris-HCl pH 8.0; 500 mM LiCl; 1% Nonidet P40; 0.1% sodium deoxycholate) and once with TE buffer (50 mM Tris-HCl, pH 8.0; 2 mM EDTA). The protein–DNA complexes were eluted with elution buffer (50 mM NaHCO_3_; 1% SDS). NaCl was added to a final concentration of 200 mM. To reverse the crosslinking, immunoprecipitated complexes were incubated 12–16 h at 65 °C in the presence of 10 µg of RNase A (Invitrogen Corp., Thermo Fisher Scientific Corp. Waltham, MA, USA). Proteins were digested with 25 μg of proteinase K for 2 h at 50 °C. DNA was recovered with the Zymo Research Kit for DNA Clean & Concentrator, and analyzed by real-time PCR (KAPA SYBR FAST, Universal qPCR kit, Supplementary Materials, Table S3). Data were processed as follows: from the Ct value obtained with the qPCR, we quantified viral DNA immunoprecipitated with respect to a standard curve prepared with the HBV genome. We then eliminated the background value obtained with the IgG control. We normalized the values with GAPDH as a loading control and with cccDNA. In the case of analyzing histone modifications, we normalized the data against the immunoprecipitated H3.

### 2.6. Epifluorescence

Approximately 3.5 × 10^5^ Huh-7 cells were seeded on 12 mm coverslips, and 24 h later, cells were transfected with plasmid containing different HBx isoform proteins fused with GFP (in vector pAcGFP) as previously reported [27,28]. Cells were washed 3 times with 1× PBS 24-h post-transfection and fixed with 4% PFA in 1× PBS for 10 min at room temperature. Cells were then washed 3 times for 5 min each with 1× PBS, and then nuclei were counterstained with 0.2 µg/mL DAPI in 1× PBS, and then washed for 5 min with water. Cells were visualized on an Olympus FSX Bio Imaging Navigator. For the quantitative analysis of the subcellular localization of the HBx-GFP isoform proteins, a total of approximately 100 positive cells for the expression of GFP constructs were analyzed from different experiments. Cells were transfected with three different amounts (High, H, Medium M, or Low L) of plasmid DNA [53], and after 24 h, coverslips were processed for fluorescence. Expression of HBx-GFP construct proteins was carefully associated to either the cytoplasm, nucleus, or nucleocytoplasmic compartments with respect to DAPI-positive nuclear staining.

## 3. Results

The nucleotide sequence of HBx coding region includes three in-frame AUG codons (AUG1, AUG2, and AUG3) (Figure 1B). Sequence alignments of HBV from the eight main viral genotypes indicated that these positions are highly conserved, with 99.6% for both AUG1 and AUG2 and 94.0% of AUG3, as reported [100]. The translation initiation process follows the scanning ribosome model in which the ribosome scans the mRNA from the 5′ end, initiating the translation at the first AUG found. However, some ribosomes may skip the first start codon (when it is in a suboptimal context) and continue scanning downstream [101,102,103,104]. This process is known as leaky scanning and is determined by the sequence context of the AUG. The Kozak consensus sequence (Figure 1B) defines the optimal sequence for translation, where the positions −3 (A/G) and +4 G are the first and second most important bases for efficient initiation, respectively. As shown in Figure 1B, the nucleotide at position −3 is in a poor context for the HBV genotype F1b isolate in both AUG1 and AUG2 cases, whereas nucleotides position +4 are in a Kozak context. In AUG3, both the −3 and +4 positions are in a poor Kozak sequence. Given that none of the three AUG codons of the HBx gene are in an optimal Kozak context, alternative translation initiation by leaky scanning could take place to translate the HBx mRNA, as suggested [75,76]. The two smaller HBx isoforms would lack the negative regulatory domain whereas the smallest HBx would contain nearly half of the transactivation domain (Figure 1A). Thus, one can anticipate that HBx isoforms could affect HBx multifunctionality. To date, the expression of the putative HBx isoforms at the protein level has not been systematically studied. We generated constructs to express the WT HBx protein and the different HBx isoforms fused at their C-terminus to the GFP reporter (Figure 1C). A construct only expressing the full-length HBx isoform (named XF) was generated by two single point mutations in the second and third AUG to GUG (methionine/valine) to prevent ATI. In the case of the HBx XS isoform protein, we initially generated a construct by starting the reading frame sequence at the third AUG. However, upon transfection, we could not detect its expression (data not shown). Instead, the construct to express the small HBx isoform (XS) was generated from the entire open reading frame but with the introduction of two single mutations: one mutation introduced a UAA in-frame stop codon at the eighth codon position (Figure 1C); the second mutation was the second AUG to GUG, to prevent ATI. We carried out Western blot analysis of cell extracts from Huh-7 cells after transfection (Figure 1D). Importantly, when the WT canonical HBx reading frame was transfected, three C-terminal tagged polypeptide isoforms were expressed, corresponding to XF, XM, and XS (Figure 1D, lane 1). We transfected the constructs to express each individual HBx isoform, and we detected each HBx polypeptide isoform, XF, XM, XS, as shown in Figure 1D, lanes 2, 3, and 4, respectively. Interestingly, comparable overexpression levels were achieved for all three HBx isoforms synthesized individually. As indicated above, overexpression of the XS isoform required expression from the complete HBx ORF, and thus this indicates that there might be important regulatory sequences for transcription/translation upstream of HBx AUG3. We further conclude that the three divergent N-terminal HBx isoforms can be expressed in Huh-7 cells from the canonical HBx reading frame.

**Figure 2:**
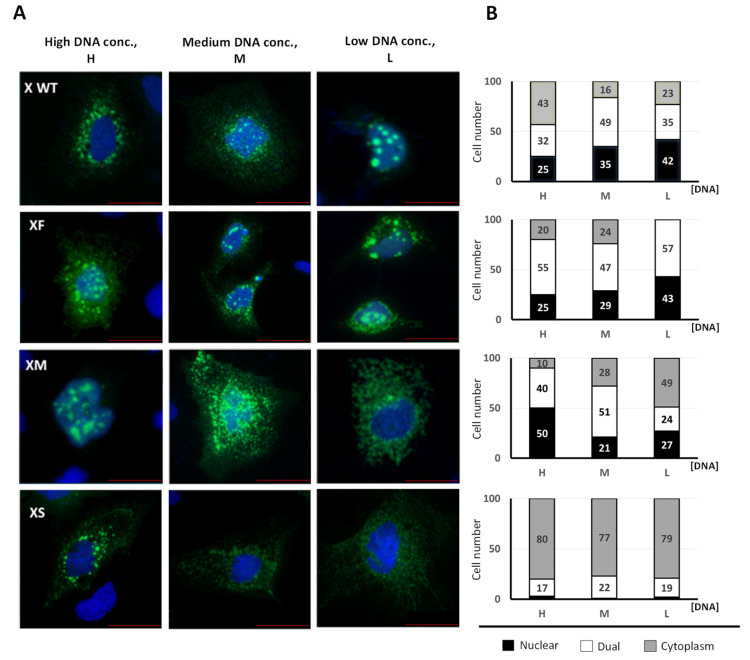
Subcellular localization of HBx canonical, and individual HBx isoforms in cultured hepatocarcinoma cells. (**A**) Fluorescence of the GFP-tagged HBx proteins in hepatoma cells transfected with high (H, left), medium (M, middle) or low (L, right) amounts of the plasmid DNA constructs [27,86,90]. Representative images of cells transfected with X WT (HBx WT), XF (HBx full-length), XM (HBx medium-length), XS (HBx short-length), each of them fused to the GFP protein are shown. Bar for scale of 50 µm. (**B**) 100 positive cells for the expression of GFP of WT or mutant HBx proteins were analyzed. Cells were all transfected with three different amounts of DNA, and after 24 h, coverslips were processed for fluorescence microscopy. Expression of HBx-GFP isoform proteins was associated with either the cytoplasm, nucleus, or nucleocytoplasmic (dual) compartments with respect to DAPI-positive nuclear staining.

The ATI mechanism can diversify the cell proteome and alter protein function and/or protein subcellular location. Since the N-terminal domain of proteins frequently contains regulatory, or destination signals, if translation is initiated from ATIs, the synthesized protein isoforms will diverge in their N-terminus domains and may be delivered to different compartments and/or display different functions [68,69,70]. We next analyzed the subcellular localization of the HBx isoforms fused to GFP (as in Figure 1C). Previous works have shown that different expression levels of HBx might influence its differential subcellular distribution [50,51,53]. We tested different amounts of DNA to achieve different levels of HBx abundance. Initially, we introduced the amino acid sequences of the HBx isoforms into the PSORT II web server to predict subcellular localization based on the protein sequence [105]. The prediction indicated that the HBx XF isoform would be predominantly a nuclear protein, the XS isoform mainly a cytoplasmic protein, and the XM isoform distributed between the nucleus and cytoplasm (Supplementary Materials, Figure S1). We next analyzed Huh-7 cells by fluorescence microscopy after transfection of the C-terminal tagged HBx isoforms (Figure 2A), where three amounts of DNA were tested.

Additionally, to quantify all forms of HBx localization, we carried out an analysis of its subcellular distribution (Figure 2B). Approximately 100 positive cells were analyzed in each case, and the expression of HBx-GFP isoforms was carefully associated with either cytoplasm, nucleus, or dual (nucleocytoplasmic compartment), with respect to DAPI-positive nuclear staining [53]. Consistent with previous results, the canonical HBx protein displayed different subcellular localizations depending on expression levels, where the protein was mainly nuclear (42%) at low levels of expression, whereas at the highest expression it was predominantly cytoplasmic (43%) (Figure 2B). Regarding the XF isoform, at low expression levels, the protein displayed a 57% dual (nucleocytoplasmic) distribution, whereas the rest (43%) was a nuclear protein [53], with no cytoplasmic localization. Increasing the abundance of the XF isoform resulted in up to 20% of the protein localized in the cytoplasm. Thus, the XF protein is probably mainly confined to both nuclear and nucleocytoplasmic compartments (Figure 2B). In the case of the HBx XM isoform, at low expression levels, the protein was predominantly (49%) cytoplasmic with equivalent levels of both nuclear and dual nucleocytoplasmic distribution. At higher expression levels, the XM isoform was predominantly retained in nucleus (50%) and dual nucleocytoplasmic compartments (40%) with minor presence in the cytoplasm (10%). Interestingly, both WT canonical HBx and the XM isoform protein behaved oppositely; whereas the canonical HBx was mainly a nuclear protein at low expression levels and predominantly cytoplasmic at the highest expression levels, the XM isoform protein was predominantly cytoplasmic at low expression levels and mainly nuclear at highest expression levels (Figure 2B). The subcellular distribution of the HBx XS isoform is intriguing. Either at a low, medium, or high levels of expression, the XS protein remained >77% cytoplasmic. For the XS isoform, a low level of dual nucleocytoplasmic distribution (around 20%) was identified across expression levels. The XS protein was hardly confined to the nucleus. Thus, the HBx XS isoform protein is a predominantly cytoplasmic protein (Figure 2B). Taken together, we have found that the individual HBx isoforms have different and overlapping subcellular locations within cells, with the XS isoform having a clear and unique cytoplasmic profile, as predicted by the bioinformatics analysis. Consequently, the different and overlapping subcellular distributions of the HBx isoform proteins might be an indication of their differential roles during HBV genome replication.

The analyses of primary sequences of the two smaller HBx isoforms indicated that HBx XM isoform lacks the negative regulatory domain but maintains the full transactivation domain, whereas the XS isoform bears only half of the transactivation domain (Figure 1A). It is known that the HBx N-terminus bears a highly conserved but unstructured and disordered region [38]. Disordered regions are protein segments that lack a defined tertiary structure but can display various interconverting states, and they frequently contain regulatory and signaling functions. Protein isoforms modify their interaction networks by selectively altering the profile of disordered regions they express [106,107,108]. In line with this, the different isoforms of p53 diverge in their content of disordered regions, and this fact correlates with the establishment/abolishment of interactions with different partners [109]. Thus, we performed detailed analyses of the predicted disordered regions of the full-length HBx protein by different web servers such as PONDR VL-XT [110,111], DisEMBL 1.5 [112], Phyre^2^ [113], PrDOS [114], and DEPICTER [115]. The primary HBx sequence was analyzed under default parameters, and results are summarized in Table 1.

Data indicated that indeed there are two main disordered regions (above the thresholds) in the HBx primary sequence, and they correlate with their presence/absence in the HBx isoforms: (i) region residues 24 to 50, including the Ser/Pro-rich region, as previously reported (Figure 1A), and (ii) region residues 85 to 104, predominantly because the segment of residues 86 to 96 have a high content of charged residues. Importantly, this disordered segment of residues 86 to 96 corresponds to the H-box, a promiscuous α-helix with which HBx binds to damage-specific DNA-binding protein 1, DDB1 (Figure 1A) [41]. The binding of HBx with DDB1 redirects the DDB1-contaning E3 ubiquitin ligase to target the structural maintenance of chromosome 5/6 complex (Smc5/6) for degradation, releasing the transcriptional repression by Smc5/6 to increase HBV gene expression [116,117]. It has been proposed that HBx adopts a functionally significant and folded conformation only upon binding to DDB1 [40]. Additionally, the region of the C-terminus of the transactivation domain is also identified as disordered (Table 1). Thus, examining the primary sequences contained by each HBx isoforms, it is apparent that they also diverge in the disordered regions present toward their N-terminus. The HBx XM isoform lacks the disordered region between residues 24 to 50 (Ser/Pro-rich region) but maintains the disordered segment between 85 to 104 (including the H-box interacting with DDB1). On the other hand, the HBx XS isoform protein is lacking the abovementioned disordered regions, and it consists of only about half of the HBx transactivation domain, but includes the α-helix BH3-like motif to interact with the anti-apoptotic factor Bcl-2 [42,43] (Figure 1A). Therefore, since the HBx isoforms display differential subcellular locations, and they bear different disordered regions and functional domains, one can anticipate that there are marked differences between the HBx isoforms that can impact their roles during viral replication.

**Figure 3:**
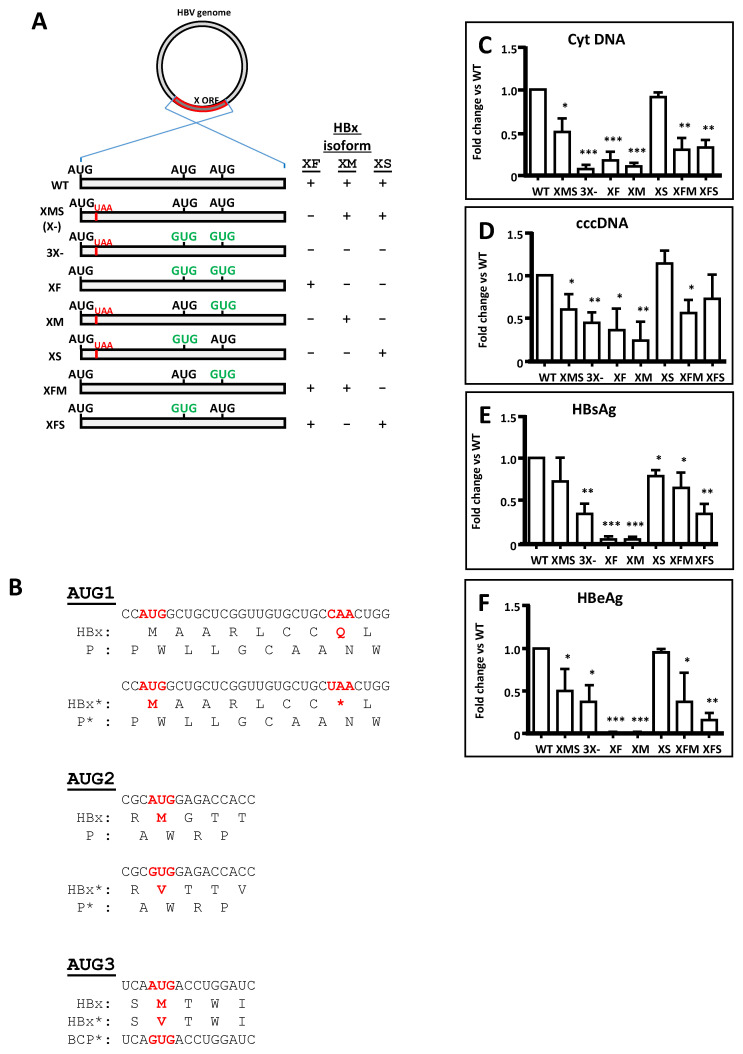
HBV DNA constructs for targeting the individual expression of the different HBx isoform proteins, and replication in HepG2 cells. (**A**) HBV DNA backbone containing HBx mutations of either individual HBx isoforms or all possible combinations of them [27,28]. To abolish the expression of HBx full-length (i.e., in the constructs XMS, 3X-, XM, XS) an early in-frame stop codon was introduced in the HBx reading frame. Additional point changes in the HBx reading frame are indicated by the GUG codon (green) replacing the corresponding AUG codon at either the second or third in-frame initiation codons. Protein expressions of each individual HBx isoforms for each HBV DNA construct are indicated in the columns to the right. (**B**) Point sequence changes on reading frames of either HBx or HBV P (polymerase) protein. Since the reading frames of HBx and P protein overlap but in a different phase (offset by one nucleotide), it is important that changes introduced into HBx reading frame do not affect HBV P protein. As shown, mutations in either AUG1 (top panel) or AUG2 (middle panel) of the HBV HBx do not affect the reading frame of the HBV P protein. HBx* and P* indicate the modified DNA and amino acid sequences. In the case of HBx AUG3 (bottom panel), it is overlapped by the basal core promoter (BCP) sequence, and the change in the sequence will introduce the indicated point mutation [118,119,120,121]. Analyses of intracellular HBV (**C**) Cyt DNA, and (**D**) cccDNA replicative intermediates after transfection of WT or mutant HBV genomes are shown, respectively. Analyses of secreted HBV (**E**) HBsAg, or (**F**) HBeAg viral antigen markers into supernatants are indicated, respectively. Results are shown as fold changes with respect to the WT HBV genome. The standard deviation was obtained from four independent experiments. *: *p* < 0.05, **: *p* < 0.01, ***: *p* < 0.001, Student’s *t*-test.

We investigated the activities of the different HBx isoforms within the HBV backbone. For this, we generated different HBx mutants directly in the HBV DNA which allowed the targeted expression of all individual HBx isoforms or their possible combinations. Figure 3A depicts the mutagenesis strategy, combined mutations, and the expected HBx isoform expression during viral replication.

Mutagenesis was designed to not alter the reading frame of the HBV P protein, as indicated in Figure 3B. The reading frames of HBx and P protein overlap but in different phase (offset by one nucleotide). Changes introduced into the in-frame initiation codons either AUG1 (top panel) or AUG2 (middle panel) of the HBV HBx do not affect the reading frame of the HBV P protein. In the case of HBx AUG3 (bottom panel), it is overlapped by the basal core promoter (BCP) sequence, and one modification to the sequence will introduce the indicated unique change (Figure 3B).

We studied HBV replication utilizing a system based on the HepG2 hepatoma cell line transfection with linear full-length HBV monomers containing cohesive ends that facilitate circularization [27,99,122]. In contrast to human hepatocellular carcinoma Huh-7 cells [96], HBx is required for HBV replication in human HepG2 cells [16]. Seventy-two hours post-transfection, we isolated HBV replicative intermediates, cytoplasmic viral core particles (cytDNA) as well as cccDNA, and quantified them via qPCR. Additionally, from the supernatants, we collected secreted markers of replication for quantification.

When the HBV genome was mutated to abolish the expression of all three HBx isoforms (3X-), the levels of cytDNA were lower than that of XMS HBV genome, as shown in Figure 3C. In the HBV mutant construct XMS, the isoform XF was abolished, and the levels of cytDNA were significantly diminished compared with that of WT HBV genome; however, viral replication was not completely prevented. Thus, the XF isoform is necessary but not essential for HBV replication, and both XM and XS polypeptides can contribute to HBV replication. Importantly, this XMS viral construct is equivalent to the viral construct that has been widely described as an HBV HBx-deficient, and that has been used as a common approach to abolish HBx expression (Figure 3A) [16,96]. In contrast, the HBV 3X- construct, designed to abolish the expression of all three HBx isoforms, reduced the detection of the cytDNA marker to baseline levels (Figure 3C). On the other hand, the mutant constructs either HBV XFM or XFS produced comparable levels of the HBV replication marker cytDNA. When we transfected the HBV genomes that only expressed individual XF or XM, cytDNA levels were low and comparable to baseline levels of the 3X- construct; thus, neither XF nor XM individually were sufficient to restore WT levels of HBV replication (Figure 3C). Importantly, cytDNA marker levels were comparable with that of the WT HBV genome when we transfected the HBV genome that only expressed the HBx XS isoform (HBV XS construct, Figure 3C). Taken together, the results indicate that, amongst the HBx isoform proteins, only the individually expressed XS isoform can restore WT levels of HBV DNA intermediates, recapitulating the levels of HBV replication.

We then analyzed the levels of the nuclear HBV intermediate cccDNA (Figure 3D). Consistent with the cytDNA results, cccDNA levels in mutant genomes XMS, 3X-, F, M, and FM were significantly lower than that of WT HBV. On the other hand, cccDNA levels in the mutant genomes XS and XFS were like those of WT HBV. Thus, in the absence of all HBx isoforms, as in the HBV 3X- construct, viral replication was maximally reduced to a baseline level. As above, the activity of the XS isoform could restore WT HBV levels of replication, as indicated by its cccDNA levels (Figure 3D).

Finally, we tested the post-transfection supernatants to detect biomarkers of acute and active viral HBV replication, such as secreted antigens HBsAg and HBeAg, respectively [123,124]. HBsAg is also a general marker for viral protein expression. Mutant genomes such as HBV XMS and 3X- both tended to diminish the levels of HBsAg antigen with respect to that of WT HBV (Figure 3E). Interestingly, the lowest values of HBsAg secretion were from the HBV XF and XM mutants, both with 5% of the WT HBV level. Mutants XFM and XFS gave secretion values of 65% and 35% of that of WT HBV, respectively, whereas the highest level of HBsAg secretion was produced by the XS isoform mutant with 80% that of WT HBV (Figure 3E). On the other hand, HBeAg is a general marker that reflects active viral replication, and results are shown in Figure 3F. Mutant genomes XF and XM gave the lowest values of HBeAg secretion with values below 1% that of WT HBV, whereas mutant genomes XFS, 3X-, XFM, and XFS gave values of 15%, 38%, 40%, and 50% that of WT HBV, respectively. The mutant genome HBV XS did not present a significantly different value of HBeAg to that of WT HBV (95% that of WT HBV, Figure 3F). Thus, consistent with the results obtained on HBV replicative intermediates, we found that the HBV XS genome had comparable levels of secreted viral antigens to the WT HBV genome. These data collectively indicate that the HBx XS isoform plays a critical role in the HBV viral cycle.

**Figure 4:**
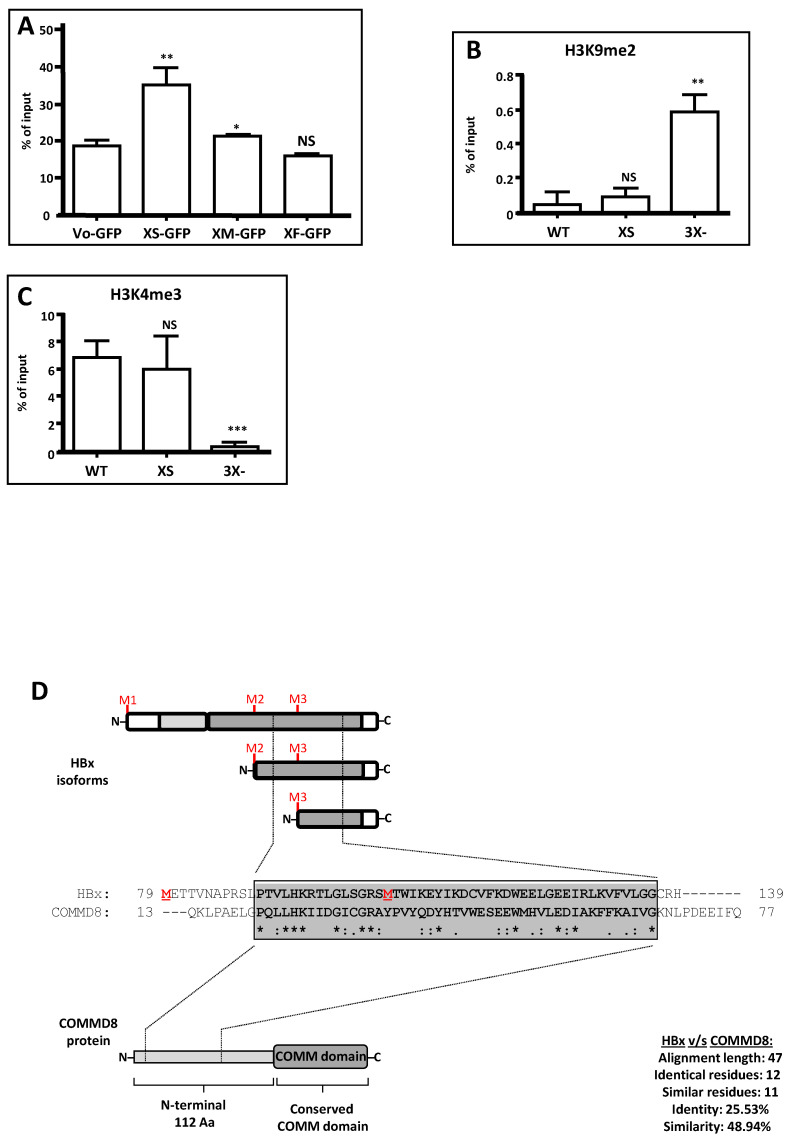
Roles and properties of the HBx XM and XS smaller isoforms. (**A**) The HBV XS isoform binds to the cccDNA and is sufficient to establish an active viral chromatin state. HepG2 cells were co-transfected with WT HBV DNA and one of the constructs, GFP-Vo (GFP empty vector), HBx XS-GFP, HBx XM-GFP or HBx XF-GFP, and the cells were analyzed by chromatin immunoprecipitation (ChIP) assays. HBx isoform binding to cccDNA was determined by ChIP analysis using GFP-conjugated Sepharose beads. Immunoprecipitated DNA was quantified by qPCR using specific primers for the core promoter. The results are expressed as % of input. The standard deviation was obtained from three PCR reactions, and the graphs are representative of three independent experiments. *: *p* < 0.05, **: *p* < 0.01, ***: *p* < 0.001, Student’s *t*-test. (**B**,**C**) Analysis of post-translational modifications of histones bound to the core viral promoter by ChIP. HepG2 cells were transfected with the viral full constructs, either WT HBV, HBV XS, or HBV 3X- genomes, and cells were analyzed 72 h later for ChIP assay. Covalent post-translational modifications on histone H3 were determined using specific antibodies H3K9me2 (**B**) and H3K4me3 (**C**). Immunoprecipitated DNA was quantified by qPCR using specific primers for the core promoter. The results are expressed as % of input and normalized against the ChIP H3 value. The standard deviation was obtained from three PCR reactions and the graphs are representative of three independent experiments. *: *p* < 0.05, **: *p* < 0.01, ***: *p* < 0.001, Student’s *t*-test. (**D**) Sequence similarity between the N-terminus of the COMMD8 protein and HBx XM isoform protein. On top, scheme showing domains and initiation codon positions M1, M2, and M3 (in red) of the three isoforms of the HBx protein, and the position of the similarity region within the isoforms. To the bottom, a scheme that depicts domain organization of COMMD8 protein, showing the conserved C-terminal COMM domain and the unique N-terminal extension (112 residues). Within its N-terminus, the similarity region is indicated. In the middle, the similarity region between HBx and COMMD8 is shown as well as amino acid residues involved in the alignment. The significant similarity region is boxed and highlighted in gray, which displays 25.53% identity, and 48.94% similarity. For these determinations, the website GenomeNet (genome.jp) was used, and the server utilized was MOTIF (sequence motif search), using the set of all databases.

HBV canonical HBx protein has been implicated in regulating different stages of cccDNA mini chromosome activities [82,85,86,87,92,93,94]. Due to its critical role in viral genome replication, we next investigated whether the XS isoform could participate in the establishment of an active HBV chromatin state, as reported for the canonical HBx protein. We first examined whether HBV X isoform proteins could individually associate with the viral cccDNA nuclear intermediate. We co-transfected HepG2 cells with the WT HBV DNA genome and the constructs expressing individually either XF, XM, or XS isoforms fused to a GFP protein. Twenty-four hours post-transfection, we performed ChIP analyses against GFP. The results are shown in Figure 4A.

As indicated, we determined that, amongst the different individual HBx isoforms, only the XS isoform was significantly bound to the viral core promoter whereas both the HBx XF and XM isoforms failed to be detected as bound to the viral promoter. Both HBx XF and XM isoforms gave baseline levels, like Vo-GFP, the empty vector (Figure 4A).

We next investigated the histone post-translational modifications associated with the viral nuclear intermediate cccDNA. For this, HepG2 cells were transfected with either the WT HBV genome, the HBV 3X- construct or the HBV XS construct. Seventy-two hours post-transfection, we performed ChIP assays against either the repressive histone modification H3K9me2 or the activating histone modification H3K4me3, with focus on the viral core promoter [21,82,84,86,87,125]. We first analyzed the H3K9me2 (Figure 4B) and observed that the mutant HBV 3X- genome was significantly enriched in H3K9me2. In contrast, both the WT HBV and the HBV XS were devoid of the repressive histone modification. This result is consistent with the described functionality of cccDNA in the absence of canonical HBx (Figure 4B). On the other hand, regarding the activating histone modification H3K4me3, we observed that, in the presence of either WT HBV or HBV XS, the viral core promoter was enriched in H3K4me3 (Figure 4C). In contrast, in the absence of any of the HBx isoforms (HBV 3X-), the viral core promoter had significantly lower levels of H3K4me3 (Figure 4C). These results are consistent with the transcriptional activity of the mutants: the WT HBV and the HBV XS genome cccDNAs were enriched in the active mark H3K4me3 and transcriptionally active (Figure 3C,F), whereas the mutant HBV 3X- genome was enriched in the histone repressive mark H3K9me2 and transcriptionally inactive (Figure 3C–F). Therefore, we conclude that XS isoform binds to the viral core promoter and is sufficient to establish an active HBV chromatin state.

Since the three divergent N-terminal HBx isoforms XF, XM, and XS correspond to naturally existing functional proteins encoded by the same gene, and since the canonical HBx (154 amino acids) shares no sequence similarity to any known protein, we utilized the primary sequence of the smaller HBx isoforms, XM (76 amino acid), and XS (50 amino acids), to search in freely accessible public databases for the identification of sequence similarities or conserved motifs within the target sequence. Using a small amino acid sequence could help to improve the significance of a found hit, if any. Whereas the database search was not successful using the smallest XS HBx isoform to look for similarities or conserved motif, intriguingly, we found an important similarity between the XM isoform and the N-terminal domain of the human COMMD8 protein [126]. The results are shown in Figure 4D. A similar sequence of 47 residues corresponding to 62% of the XM isoform primary sequence and to 42% of the N-terminal domain of the COMMD8 protein was determined by the alignment, with no gaps (Figure 4D). Overall, the 47-residue alignment displays 25.5% identity and 48.9% similarity in the segment of similarity, and the positions of these residues show up to 72% of conservation across different HBV genotypes. Human COMMD8 belongs to the copper metabolism MURR1 domain-containing (COMMD) protein family, which is composed of 10 members (COMMD1–10), and is highly conserved among multicellular eukaryotic organisms [126,127]. COMMD proteins have no described enzymatic activity, and they probably act by protein–protein interactions and as scaffold proteins favoring macromolecular protein complex formation. However, COMMD proteins have been related to the regulation of transcription factors such as NF-κB, and some of the members of the family are suppressed in tumor cells, suggesting several antitumor roles [126,128,129,130]. COMMD members associate to distinct Cullin proteins, acting as core scaffolds and along with RING box proteins to form the heterodimeric Cullin–RING ligases (CRLs) [131]. Thus, this interesting and novel finding seems to indicate that HBx might be somehow distantly related to the COMMD8 protein.

**Figure 5:**
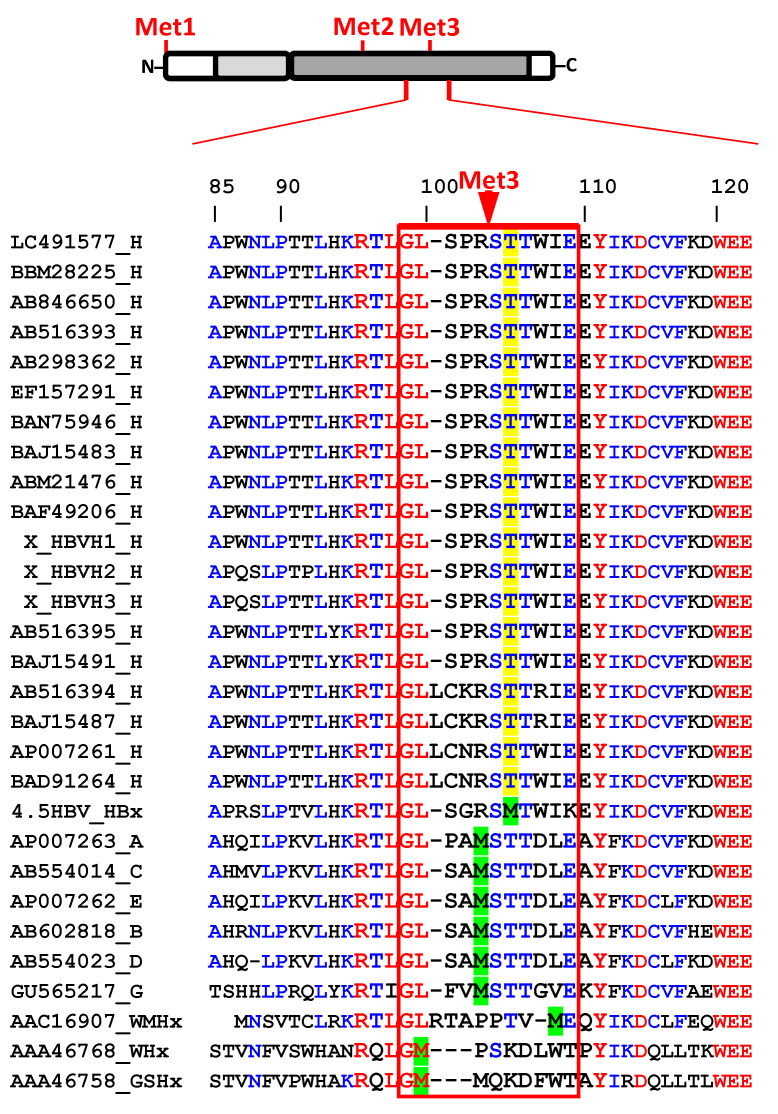
HBx XS small isoform is not expressed in HBV genotype H isolates. Multiple alignment of primary sequences of HBx proteins from all main human HBV genotypes, and chronic hepatitis B-type viruses from other organisms, such as woolly monkey hepatitis virus X protein (HMHx), woodchuck hepatitis virus X protein (WHx), and ground squirrel hepatitis virus X protein (GSHx) using multiple sequence comparison by log-expectation, the MUSCLE web server [132,133]. The alignment is magnified surrounding the Met3 position, between residues 85 to 122 of canonical HBx protein as indicated on top of the figure. HBx protein individual accession numbers are indicated in the left column, followed by the corresponding HBV genotype of each isolate or organism. The isolate 4.5HBV_HBx corresponds to our lab working HBV HBx genotype F1b isolate (GenBank: AIL83994.1) [27,28]. Individual residues across samples are colored as conserved (red), semi-conserved (blue) or non-conserved (black). Highlighted positions in bold and green indicate position of HBx Met3 in most of genotypes. Instead, highlighted position in bold and yellow indicate the corresponding conserved position of HBx Thr3, in HBV genotype H isolates, implicating the absence of Met3 in these isolates.

Finally, we further subjected HBx isoform sequences to more profound analyses. Whereas it is known that within HBx AUG1 and AUG2 are highly conserved (99.6%) across genotypes, AUG3 has been reported to display only up to 94% conservation between genotypes [100]. We recovered twenty sequences (from full-length viral isolates) of HBx protein from HBV genotype H isolates from public databases to contrast them with those representatives from other viral genotypes. The results are shown in Figure 5, where we performed sequence alignment of the full-length HBx proteins, but we magnified the sequences surrounding AUG3.

Whereas HBx AUG3 shows conservation in woodchuck hepatitis virus (WHV), woolly monkey hepatitis virus (WMHV), ground squirrel hepatitis virus (GSHV), and in human isolates that are also able to cause chronic infections such as genotypes A to G, surprisingly, all isolates from HBV genotype H are lacking AUG3. As shown in Figure 5, Met3 is absent in all sample of genotype H, and instead, a threonine is placed at that position, which is also conserved in that genotype. This finding signifies that HBx XS, the smallest isoform, is not naturally produced throughout HBV genotype H infection. Unlike infections with other genotypes, infections by HBV genotype H are characterized by few cases of acute and chronic liver diseases, a low prevalence of HCC, low endemicity, and low viral load. Individuals infected with HBV genotype H are asymptomatic without clinical manifestations of liver disease [12,13,14,15]. The molecular determinants of the different clinical outcomes between HBV genotype H and the other genotypes are unknown. Additionally, HBV genotype H isolates replicate at low level in cultured human hepatocarcinoma cells [134]. In line with this, we have shown that an HBV construct in which the expression of the HBx XS isoform has been abolished (construct HBV XFM, subgenotype Figure 1B, Figure 3A,C–F), is severely impaired for genome replication. Thus, whether the natural genetic abolishment of the HBV XS isoform is one of the causes of the different clinical outcome of HBV genotype H infections remains to be investigated.

## 4. Discussion

Our work has demonstrated that the expression of the HBV HBx reading frame generates not a unique and canonical polypeptide, but rather three divergent N-terminal HBx isoforms, named XF (Aa: 1–154), XM (Aa: 79–154), and XS (Aa: 105–154) (Figure 1). We have also shown that the canonical HBx protein and the individual isoforms displayed different subcellular localization in human hepatocarcinoma cells. Our results suggest that the XF protein isoform has no preferential subcellular localization, and thus possibly, during infection, the interaction with the smaller protein isoforms might direct the localization of XF specifically to nuclear and/or cytoplasmic compartments (Figure 2). Consistent with our assumption, both HBx XM and XS isoforms, when they are expressed from a shorter HBx transcript, displayed mainly a cytoplasmic distribution [70]. The different and overlapping subcellular localizations of the HBx isoforms reflects their different and multiple roles during HBV genome replication.

Directly within the HBV backbone, we have genetically dissected the HBx reading frame to target the individual expression of each protein isoforms, either the XF full-length, the XM or the XS or all combinations of them to assess their roles during HBV genome replication (Figure 3). As a negative control, we designed an HBV construct bearing all the mutations required to completely abolish HBx isoforms expression; HBV 3X- (Figure 3). Importantly, with this mutant, viral replication was significantly reduced to 8.6% of the WT virus, and consistently, viral cccDNA was enriched in the repressive histone mark H3K9me2. In contrast with the HBV XMS construct which is equivalent to the previously known HBV HBx-deficient construct, the replication (cytDNA) was reduced only up to 50% of the WT HBV. Both XM and XS protein isoforms are expressed from this construct (HBV XMS), and they both contribute to replication, although with up to 50% of WT HBV. Consistent results were also obtained by examining the corresponding HBV secretion markers from these constructs (Figure 3) Therefore, it is necessary to highlight that the truly HBV HBx-deficient construct is HBV 3X-. The HBV constructs XF, XM, XFM, and XFS are still deficient for viral DNA synthesis, and none of them can restore full replication. We do not know the reason of this, but we can speculate that the N-terminal negative regulatory domain, displaying transrepressor activity, would limit the protein activity of XF, the individual full-length HBx isoform. However, the lack of replication activity of the XM isoform seems to indicate that the absence of the N-terminal negative regulatory domain is not enough to restore replication. Our results are consistent with those of others who found an HBx Aa 79 to 154 construct inactive for secretion markers in HBV infected primary human hepatocytes [40]. Moreover, we found that only the HBx XS isoform (henceforward referred to as “mini-HBx”) was significantly bound to the viral core promoter in the cccDNA to activate transcription, whereas individually expressed isoforms, either XF or XM, failed to bind to the viral promoter. Thus, the construct HBV XS, which only expresses the “mini-HBx” isoform, could achieve WT levels of HBV replication (cytDNA), and these levels were consistent with the secretion of HBV markers HBsAg and HBeAg. Additionally, we also showed that the viral cccDNA derived from the HBV construct expressing only the “mini-HBx” isoform was enriched in the active histone mark H3K4me3 and therefore it is transcriptionally active, consistent with previous results about cccDNA regulation [21,82,84,86,87]. Collectively, our results seem to indicate that WT levels of HBV replication requires the simultaneous, and regulated expression of all the HBx isoforms within cells. Otherwise, only the individually expressed “mini-HBx” could overcome the host-imposed transcriptional silencing, and it possibly acts as a recruiting factor to locally modify the chromatin of viral cccDNA promoters. The engagement of the HBx XF full-length isoform protein, previously detected as bound to the viral cccDNA sites, should also take place. On the other hand, two regions, residues 88 to 154, and residues 132 to 139, both critical for the stimulation of HBV transcription and replication, have been previously identified both in vitro and in vivo [19,25,32], and are consistent with the C-terminal region covered by the “mini-HBx”. Therefore, our systematic HBV mutagenesis to target the expression of the HBx proteins unveils the roles of the different isoforms by individually dissecting their crucial activities, particularly the HBV “mini-HBx” isoform (HBx region residues 79 to 154).

Considering that the “mini-HBx” isoform displayed not only a predominantly cytoplasmic localization but also a minor nuclear localization (Figure 2), and it does bear nearly half of the transactivation domain, it is possible to envision that this critical isoform can act on genome replication in two different ways [51]; (i) in the nucleus, by its minor but perhaps, crucial active amounts and through the nuclear transactivation domain (residues 120–140) [31,35] since this region fully retains the capacity to interact with the transcriptional and chromatin modification machinery (Supplementary Materials, Table S4), and to bind cccDNA (Figure 4A); or (ii) In the cytoplasm, through the “mini-HBx” activation of the Src family of tyrosine kinases to enhance viral replication [17,56,135,136]. Alternatively, the “mini-HBx” can activate genome replication through its BH3-like motif which folds into a short α-helix that interacts with the anti-apoptotic factor Bcl-2 [43,137,138,139]. This interaction can trigger the increase of calcium in the cytosol, which is required for HBV viral replication [42,43,140,141]. Importantly, an HBx-BH3-like peptide could restore HBV replication in HepG2 cells transfected with an HBV HBx-deficient construct, reflecting the crucial functional role of this short α-helix, BH3-like motif to stimulate viral replication [43]. Regarding the subcellular localization of the canonical HBx, Cha et al., proposed that, during the early stages of chronic HBV infection, the HBx protein would preferentially locate to the nucleus. Throughout viral persistence, HBx levels increase through genetic alterations or inflammatory effects, and so the late phases of a chronic infection can lead to the cytoplasmic accumulation of HBx. Consequently, sustained expression of HBx during the late phase of chronic infection may be associated with disease progression [51]. If this proposal reflects the pathway of either the canonical HBx or the “mini-HBx” during long-term infection, remains to be determined. On the other hand, we have provided strong evidence that the “mini-HBx” isoform protein expression is naturally abolished in HBV from genotype H (Figure 5), whose infections cause less severe clinical outcomes compared with other HBV genotypes. Together, these findings seem to further highlight not only the relevance of the subcellular localization of the “mini-HBx” protein, but also the possible involvement of each HBx isoform, in HBV pathogenesis, and chronicity.

Back in 1992, Kwee et al. assayed, in heterologous systems, the trans-activation activity of HBx isoforms (XF, XM, and XS) contained in expression plasmids on class II (transcribed by RNA polymerase II) and class III (transcribed by RNA polymerase III) promoters [75]. They found that each HBx isoform, expressed individually, was capable of transactivating class III promoters. However, the class II promoter showed different requirements for transactivation by the HBx isoforms. Significantly, the SV40 Enh/Early promoter was trans-activated only in conditions of co-expression of both the XF and “mini-HBx” isoforms. This seems to indicate a higher level of regulation between the different HBx isoforms, particularly between both the XF and “mini-HBx” isoforms. In the case of the NF-κB promoter, only the full-length XF protein displayed activation. Therefore, different activities from different HBx isoforms can transcriptionally regulate class II promoters, and thus HBx protein isoforms can be functionally divergent or act as regulatory polypeptides [75]. Importantly, the HBx highly conserved region E-domain (residues 120–140) which is essential for transactivation [31], can also promote the physical association of different molecular forms of HBx [142]. Since the E-domain is common to all the individual isoforms, this finding seems to suggest that the isoforms might associate through their C-terminus, possibly forming functional protein complexes. This would add a higher and more complex level of HBx isoform regulation. On the other hand, Leach et al., investigated the effects of HBx and HBx isoforms on the expression of cyclin kinase inhibitor (CKI), p21, and p27 polypeptides, and its impact on cellular proliferation [76]. They found that low levels of both XF and XM enhanced, and then at higher levels suppressed the expression of p21 and p27. Moreover, low concentrations of both XF and XM resulted in a decrease in cellular DNA synthesis. The “mini-HBx” was increasingly stimulatory with dose dependence for both promoters, and for reduction in cell DNA synthesis [76]. Thus, the possible functional cross-regulation between the different HBx isoforms is a fundamental open question.

Alternative transcription initiation is a mechanism by which one gene has multiple transcription start sites. The transcription of the reading frame can start from one of several transcription start sites, and from different alternative core promoters [143,144]. Not much is known about alternative transcription initiation in the regulation of HBx expression. Shorter gene transcripts with heterogeneous 5′ ends upstream of the AUG2 codon of the HBx reading frame were identified and those transcripts synthesized proteins [71]. However, the studies did not define how many different HBx isoforms were expressed. Recently, a different and smaller transcript with a peak between the AUG1 and AUG2 sequence was detected by single-nucleotide resolution of HBV transcripts. This transcript was different to that the canonical HBx mRNA [100]. Consistently, the ChIP-Seq approach on viral cccDNA found a peak of histone marks that correlated to active promoters (H3K4me3 and H3K27ac) in the middle of the HBx reading frame, which might be associated with a novel transcription initiation site between the AUG1 and AUG2 sequences [145]. Therefore, there is some evidence suggesting that alternative transcription initiation might also play a role in the complex HBx regulation. The existence of smaller HBx isoforms in vitro suggests that the expression of HBx gene products might be highly regulated at either the transcriptional level, by alternative transcription initiation, or at the posttranscriptional level, by alternative translation initiation.

We found a significant sequence similarity between the HBx and the N-terminal domain of the human COMMD8 protein (Figure 4D). The COMMD8 protein (183 Aa, 21 kDa) is constituted by a non-conserved N-terminal domain of 112 Aa, and by a C-terminal COMM domain of 71 Aa. The COMMD8 protein localizes to both nucleus and cytoplasm, and it is expressed in liver tissue [126,127]. COMMD8 protein functions have not been entirely defined. It is known that COMMD8 can interact with endogenous Cullins (CUL1, CUL3, CUL4B, and CUL5), and modulate Cullin–RING E3 ubiquitin ligase complexes [126,130]. COMMD8 also interacts with NF-κB subunits [126,130], regulating NF-κB activity. COMMD8 can also bind to the coiled-coil domain containing 22 (CCDC22 protein), which can promote the ubiquitination and degradation of the NF-κB inhibitor, IκBα [146]. The expression of COMMD8 protein negatively correlates with patient overall survival [147]. On the other hand, HBx can interact with Cullin 4–RING E3 ubiquitin ligase complex (CRL4 complex) through its interaction with DDB1 protein (CRL4-adaptor protein) [41,148]. As a CRL4 adaptor, DDB1 protein facilitates its function via interactions with DCAF (DDB1 Cullin-associated factor) receptors which associate specific substrates to the CRL4 for ubiquitination, which can result in proteasomal destruction [149]. In the case of viral HBx, the substrate is Smc5/6, as indicated earlier. Overall, it has been proposed that HBx can function as a viral DCAF since it contains a DDB1-binding motif common to other DCAF proteins [41]. Other viral proteins with DCAF activity for different viruses have been also described [150,151,152]. Additionally, HBx protein can activate NF-κB activity, and NF-κB signaling has been associated with pathogenesis and HCC [153,154]. Thus, both COMMD8 and HBV HBx proteins display functional roles in the protein degradation pathway. Furthermore, the high relevance of the COMMD8-similarity region within the transactivation domain of HBx (residues 89 to 136) is revealed by the significant number of protein–protein interactions taking place either within or nearby the similarity sequence, as shown in Table S4 (Supplementary Materials). Therefore, these interesting and novel findings regarding sequence similarity with the COMMD8 protein will further facilitate extensive studies on the phylogenetic origin of HBV X protein.

Finally, in the literature, the number of examples of newly identified divergent N-terminal protein isoforms is expanding, and from diverse organisms such as mammals, plants, and yeast [68,70]. In plants, ATP sulfurylase isoforms display a dual localization in plastids and cytosol [155]. In the yeast *Saccharomyces cerevisiae*, the protein isoforms of glutathione reductase are targeted to either mitochondrial or cytoplasm compartments [156]. In mammals, the synthesis of protein isoforms occurs with p53 [157], caspase-2 [158], glucocorticoid receptor [159], mitochondrial antiviral-signaling protein (MAVS) [160,161], Runt-related transcription factor 1 RUNX1 [162], tumor suppressor PTEN [163], stress-activated protein kinase MK2 [164], human c-myc proto-oncogene [165], and polypyrimidine tract binding protein (PTBP1) [166]. In the case of HBV X protein, the current identification and functional characterization of its isoforms will shift the HBx paradigm by changing the concept of HBx from being a unique canonical, and multifunctional protein into becoming different HBx isoforms, each carrying out different overlapping functions at different subcellular locations during HBV genome replication. Significantly, our current work, unveils new crucial HBV targets to study for potential antiviral research, and human virus pathogenesis.

## Figures and Tables

**Table 1:** Analyses of HBx disordered regions.

WEB TOOL ^2^	Predicted Disordered HBx Regions ^1^			
	Region 1–78		Region 79–104	Region 105–154
PONDR VL-XT ^3^	26–52		85–96	–
DisEMBL 1.5 ^4^	24–50		–	–
Phyre ^2 5^	1–7	25–52	98–105	147–154
PrDOS ^6^	1–3	26–44	–	150–154
Depicter ^7^	1–5	15–78	–	–

^1^ HBx GenBank: AIL83994.1; ^2^ Primary HBx sequence analyzed under default server parameters; ^3^ PONDR VL-XT, http://www.pondr.com/ (accessed on 10 November 2021); ^4^ DisEMBL, http://dis.embl.de/ (accessed on 10 November 2021); ^5^ Phyre ^2^, http://www.sbg.bio.ic.ac.uk/phyre2/html/page.cgi?id=index (accessed on 10 November 2021); ^6^ PrDOS, https://prdos.hgc.jp/cgi-bin/top.cgi (accessed on 10 November 2021); ^7^ DEPICTER, http://biomine.cs.vcu.edu/servers/DEPICTER/ (accessed on 10 November 2021).

## Data Availability

The datasets used and/or analyzed during the current study are available from the corresponding authors on reasonable request.

## Supplementary Materials

The following material is available online at https://www.mdpi.com/article/10.3390/biomedicines9111701/s1: Table S1. Primers utilized to clone HBx isoforms for expression (vector pAcGFP); Table S2. Primers utilized in the site-directed mutagenesis of HBx; Table S3. Primers utilized in the detection of HBV DNA intermediates; Figure S1. PSORT II predicted subcellular localization of HBx isoforms; Table S4. Mapped HBx protein–protein interactions involving COMMD8-similarity region.
