# Supplementary material for: Canonical and Divergent N-Terminal HBx Isoform Proteins Unveiled: Characteristics and Roles during HBV Replication
Source: Biomedicines. 2021 Nov 16;9(11):1701. doi: 10.3390/biomedicines9111701 (PMC8616016; doi:10.3390/biomedicines9111701)
Supplement: Supplementary file 1 [file biomedicines-09-01701-s001.zip › biomedicines-1449250-supplementary.pdf]

Table S1. Primers utilized to clone HBx isoforms for expression (vector pAcGFP)	
HBX WT Forward	5′-GATCGATCGATCAAGCTTATGGCTGCTCGGTTGTGCTG-3′
HBX WT Reverse	5′-GATCGATCGATCGGTACCGTGGCAGAGGTGAAAAAGTTGCA-3′
HBX F Forward	5′-GATCGATCGATCAAGCTTATGGCTGCTCGGTTGTGCTG-3′
HBX F Reverse	5′-GATCGATCGATCGGTACCGTGGCAGAGGTGAAAAAGTTGCA-3′
HBX M Forward	5′-GATCGATCGATCGATCAAGCTTATGGAGACCACCGTGAACGC-3′
HBX M Reverse	5′-GATCGATCGATCGGTACCGTGGCAGAGGTGAAAAAGTTGCA-3′
HBX S Forward	5′-GATCGATCGATCGATCAAGCTTATGACCTGGATCAAAGAATA-3′
HBX S Reverse	5′-GATCGATCGATCGGTACCGTGGCAGAGGTGAAAAAGTTGCA-3′

Table S2. Primers utilized in the site-directed mutagenesis of HBx	
Q8stop Forward	5'-CTCGGTTGTGCTGCTAACTGGATCCTGCG-3'
Q8stop Reverse	5'-CGCAGGATCCAGTTAGCAGCACAACCGAG-3'
M79V Forward	5'-CTCTGCACGTCGCGTGGAGACCACCGT-3'
M79V Reverse	5'-ACGGTGGTCTCCACGCGACGTGCAGAG-3'
M105V Forward	5′-GACTTTCAGGAAGGTCAGTCACCTGGATCAAAGAATA-3'
M105V Reverse	5′-TATTCTTTGATCCAGGTGACTGACCTTCCTGAAAGTC-3'

Table S3. Primers utilized in the detection of HBV DNA intermediates	
BCP Forward	5′-GGAAGGTCAATGACCTGGATC-3′
BCP Reverse	5′-ATGCCTACAGCCTCCTAATAC-3′
cccDNA Forward	5′-ACTCTTGGACTTTCAGGAAGG-3′
cccDNA Reverse	5′-TCTTTATAAGGGTCAATGTCCAT-3′

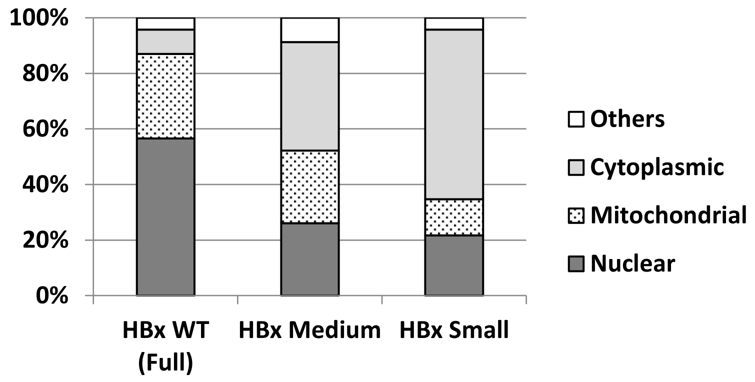

Figure S1. PSORT II predicted subcellular localization of HBx isoforms.
HBV HBx genotype F1b (Genebank KM233681.1) primary sequences of Full-length (154 amino acids), Medium-length (amino acids region 79 to 154) or Small-length (amino acids region 105 to 154) isoform proteins were introduced into the server, and the subcellular localization results were plotted.

Table S4. Mapped HBx protein-protein interactions involving COMMD8-similarity region

**Mapped HBx PPIs involved in transcription, and chromatin regulation**			
**HBx residues region**	**Binding partner**	**Properties**	**PMID**
47 to 133	E2F transcription factor 1	Role in control of the cell cycle and action of tumor suppressor proteins	11244564
51 to 154	NF-AT1, nuclear factor of activated T cells 2	Transcription factor with roles in development and function of the immune system	12202232
51 to 154	ASC-2, Activating signal cointegrator 2	Transcriptional coactivator for nuclear hormone receptors	14578865
52 to 102	SUV39H1, histone-lysine N-methyltransferase	Tri-methylates lysine 9 of histone H3, resulting in transcriptional silencing	32514521
57 to 154	HIF-1α	Transcriptional regulation of angiogenic factors	15527772
73 to 120	Estrogen receptor α	Transcriptional regulation of target genes	16757575
81 to 120	BAF155 complex subunit	Chromatin remodeling, member of SWI/SNF family, display helicase and ATPase activities to regulate transcription by altering chromatin	31533543
100 to 120	FXR, farnesoid X nuclear receptor	Ligand-activated transcription factor	28102638
110 to 143	TFIIH, general transcription factor	DNA helicase components ERCC2 and ERCC3	8855220<br>21375739<br>8670843
111 to 114, YFKD motif	NF-Kappa-B Subunit p65, RELA	Nuclear transcription factor	20010875<br>21459755
112 to 133	Id1 and Id3, inhibitor of differentiation 1 and 3 proteins	HLH proteins, transcriptional regulators	32305567
131 to 134, VFVL motif	c-Myc, proto-oncogene	BHLH transcription factor, roles in cell cycle progression, apoptosis and cellular transformation	26165841
131 to 154	SMYD3 protein	Histone methyltransferase SET and MYND domain-containing protein 3	26616333
**Mapped HBx PPIs with organelle-resident proteins**			
**HBx residues region**	**Binding partner**	**Properties**	**PMID**
72 to 117	COXIII, mitochondrially encoded cytochrome C oxidase III	Enzyme of the mitochondrial electron transport chain which drives oxidative phosphorylation	25483779
73 to 154	MAVS, mitochondrial antiviral signaling protein	Required in the virus-triggered beta interferon signaling pathways	20554965
88 to 117	Hsp60, mitochondrial chaperonin	Role in folding and assembly of newly imported proteins in the mitochondria	15120623
110 to 154	Hepatocystin/80K-H	Beta-subunit of glucosidase II, an N-linked glycan-processing enzyme in the endoplasmic reticulum.	23644164
137 to 140, CRHK motif	HBXIP	Late endosomal/lysosomal adaptor, MAPK and MTOR activator 5	18032378
**Mapped HBx PPIs with cell proteins of other processes**			
**HBx residues region**	**Binding partner**	**Properties**	**PMID**
51 to 154	hBubR1, BUB1 mitotic checkpoint	Kinase involved in spindle checkpoint function	18193091<br>23536579
84 to 144	Proteasome complex	Protein complexes that degrade proteins	11710562
88 to 100	UV-DDB 1, damage specific DNA binding protein 1	Role in DNA repair and protein ubiquitination	19966799
K91, K95, K113, K118, K130	Ubiquitination sites	Ubiquitination targets protein for destruction	18155658
100 to 128	TRIM14, tripartite motif protein	Role in the innate immune defense against viruses and bacteria	30150992
102 to 136	p53	Tumor suppressor	9371515
113 to 135	Bcl-2 and Bcl-xl	Anti-apoptotic proteins of the Bcl-1 family	26858413
